# Weevil endosymbiont dynamics is associated with a clamping of immunity

**DOI:** 10.1186/s12864-015-2048-5

**Published:** 2015-10-19

**Authors:** Florent Masson, Yves Moné, Aurélien Vigneron, Agnès Vallier, Nicolas Parisot, Carole Vincent-Monégat, Séverine Balmand, Marie-Christine Carpentier, Anna Zaidman-Rémy, Abdelaziz Heddi

**Affiliations:** Université de Lyon, INSA-Lyon, INRA, UMR203 BF2I, Biologie Fonctionnelle Insectes et Interactions, F-69621 Villeurbanne, France; Université de Lyon, CNRS, UMR5558 LBBE, Laboratoire de Biométrie et de Biologie Évolutive, F-69621 Villeurbanne, France; Present address: Université Montpellier 2, INRA, UMR 1333 DGIMI, Diversité, Génomes et Interactions Micro-Organismes Insectes, F-34095 Montpellier, France; Present address: Department of Epidemiology and Public Health, Division of Epidemiology of Microbial Diseases, Yale University School of Medicine, New Haven, CT USA

**Keywords:** Symbiosis, Transcriptome, Autophagy, Immunity, Antimicrobial peptide, *Sitophilus*

## Abstract

**Background:**

Insects subsisting on nutritionally unbalanced diets have evolved long-term mutualistic relationships with intracellular symbiotic bacteria (endosymbionts). The endosymbiont population load undergoes changes along with insect development. In the cereal weevil *Sitophilus oryzae*, the midgut endosymbionts *Sodalis pierantonius* drastically multiply following adult metamorphosis and rapidly decline until total elimination when the insect achieves its cuticle synthesis. Whilst symbiont load was shown to timely meet insect metabolic needs, little is known about the host molecular and immune processes underlying this dynamics.

**Methods:**

We performed RNA sequencing analysis on weevil midguts at three representative phases of the endosymbiont dynamics (i.e. increase, climax and decrease). To screen genes which transcriptional changes are specifically related to symbiont dynamics and not to the intrinsic development of the midgut, we further have monitored by RT-qPCR sixteen gene transcript levels in symbiotic and artificially non-symbiotic (aposymbiotic) weevils. We also localized the endosymbionts during the elimination process by fluorescence microscopy.

**Results:**

Functional analysis of the host differentially expressed genes by RNA sequencing showed that the main transcriptional changes occur during endosymbiont growth phase and affect cell proliferation, apoptosis, autophagy, phagocytosis, and metabolism of fatty acids and nucleic acids. We also showed that symbiont dynamics alters the expression of several genes involved in insect development. Our results strengthened the implication of apoptosis and autophagy processes in symbiont elimination and recycling. Remarkably, apart from the coleoptericin A that is known to target endosymbionts and controls their division and location, no gene coding antimicrobial peptide was upregulated during the symbiont growth and elimination phases.

**Conclusion:**

We show that endosymbiont dynamics parallels numerous transcriptional changes in weevil developing adults and affects several biological processes, including metabolism and development. It also triggers cell apoptosis, autophagy and gut epithelial cell swelling and delamination. Strikingly, immunity is repressed during the whole process, presumably avoiding tissue inflammation and allowing insects to optimize nutrient recovery from recycled endosymbiont.

**Electronic supplementary material:**

The online version of this article (doi:10.1186/s12864-015-2048-5) contains supplementary material, which is available to authorized users.

## Background

Insects colonize various nutritional niches, including highly unbalanced media like plant sap or cereal grains, which are rich in carbohydrates but lack components essential for animal growth and reproduction. Some species have evolved permanent relationships with intracellular bacteria (endosymbionts) that complement their diet, hence increasing their fitness and invasive power by allowing the host to overcome the environment nutritional deficiency [1, 2]. Endosymbionts are housed inside bacteriocyte cells, i.e. specialized host cells that isolate them from the humoral and cellular immune responses and ensure their control and strict localization through adapted local immune response and regulation [3, 4].

Endosymbiosis is a dynamic process, and endosymbiont density was shown to vary in many insect models. In aphids, the primary endosymbiont *Buchnera aphidicola* population increases during the first stages of development and then gradually decreases with host senescence [5, 6]. *B. aphidicola* population is also regulated by nutrient: the bacterial density is positively correlated with nitrogen availability in the insect diet [7]. In the carpenter ant *Camponotus floridanus,* the primary endosymbiont *Blochmania floridanus* density undergoes drastic changes during the host developmental process, increasing during metamorphosis, and decreasing in the adult. This dynamics has been proposed to be the result of an adaptive mechanism allowing the adjustment of the symbiont density according to the varying metabolic needs of the host [8]. Endosymbiont elimination is tightly regulated, as evidenced in the mealybug *Planococcus*, which harbors two different endosymbionts. In this association, bacteriocytes degenerate during pupation and both endosymbiont populations are eliminated, each with a separate kinetics, highlighting a specificity of the elimination process [9]. However, while symbiont density dynamics is a well-documented phenomenon, little is known about how this dynamic impacts host developmental processes and adaptive features, and which cellular and immune mechanisms of the host underlie the symbiont fluctuations [10].

In this work, we aimed at deciphering the cellular and molecular processes involved in the control of endosymbiotic population density. To address this question, we used the association of the cereal weevil, *Sitophilus oryzae*, with the γ-proteobacterium *Sodalis pierantonius* [11, 12]*.* Weevil bacteriocytes are grouped together to form the bacteriome organ, where the endosymbionts are located. In the larval stages, the bacteriome is a C-shaped organ located at the junction between the foregut and the midgut, and undergoes very little change through the larval life of the insect. The larval stages and the pupae take place inside cereal grains, where females lay their eggs. Following metamorphosis, the imago remains inside the grain for three days before emerging. During the first days of imaginal life*,* numerous bacteriomes differentiate and grow in size at the apex of the midgut mesenteric caeca, and the endosymbiotic population undergoes concomitantly a drastic increase in number [13]. This increase takes place from the first to the sixth day after the Ultimate Insect Molting (UIM), and helps the host to face a critical need in phenylalanine and tyrosine that are required for the adult cuticle synthesis during this phase of insect development [13]. A hard and thick cuticle is an adaptive trait for coleopteran species that grants them a physical protection as well as a barrier against pathogens [14]. Once the cuticle synthesis is over, the nutritional complementation by the endosymbiont becomes less critical for the host. The endosymbiotic population decreases and bacteriocytes are cleared from the gut from the seventh to the fifteenth day after UIM. Endosymbionts are recycled through apoptosis and autophagy. It was speculated that symbiont recycling allows the host to recover part of the energy and nutrients invested during bacterial multiplication and cuticle formation [13].

To unravel the cellular and molecular processes involved in *S. pierantonius* dynamics, we have performed a Next-Generation Sequencing (NGS) of *S. oryzae* midgut transcriptome during the endosymbiont burst, climax and elimination. We have identified differentially active pathways between these key points of endosymbiont dynamics through a KEGG-based analysis (Kyoto Encyclopedia of Genes and Genomes). We then compared the kinetics of expression of representative genes of each pathway between the symbiotic strain and an artificially endosymbiont-depleted strain (aposymbiotic) to highlight transcriptomic regulations that are specifically connected with endosymbiont dynamics. Our results suggest a connection between endosymbiont dynamics and insect development through the Wnt/wingless pathway and hormonal signaling. They also confirm the involvement of autophagy and apoptosis in symbiont recycling and show that these processes are transcriptionally set-up during the symbiotic burst already. Remarkably, this work highlights a clamping of the immune signaling in the midgut while endosymbionts are being digested. We speculate that this immune repression is an adaptive mechanism that allows the host to maximize nutrient recovery from the symbiont digestion by avoiding the release of a resource-expensive immune response that would interfere with endosymbiont digestion.

## Methods

### Biological material

*Sitophilus oryzae* weevils are reared on wheat grains at 27.5 °C and at 70 % relative humidity. The Bouriz strain was chosen in this work because it is free of any facultative symbionts, including *Wolbachia*, and it harbors only *S. pierantonius*. Aposymbiotic insects were obtained as previously described [15]. The aposymbiotic status was confirmed by PCR and histology. Weevil larvae and pupae grow naturally inside the wheat grain.

Insect guts were dissected from fourth instar larvae in Buffer A (25 nM KCl, 10 nM MgCl2, 250 nM Sucrose, 35 nM Tris/HCl, pH = 7.5). Tissues were pooled and stored at −80 °C, prior to RNA extraction.

### RNA isolation and libraries construction for RNAseq

RNAseq libraries were constructed from symbiotic insect guts at day 1, day 6 and day 9 after UIM (D1, D6 and D9 respectively). Total RNA from 20 guts was extracted with Trizol reagent (Invitrogen) following the manufacturer’s instructions. RNA was incubated with 1 U/μg of RQ1 RNase-free DNase for 30 min, at 37 °C then purified with RNeasy micro kit (QIAGEN) clean-up protocol. Two independent biological replicates of 1 μg of RNA sample from D1, D6 and D9 guts were provided to ProfileXpert (Lyon, France) and sequenced on an Illumina HiSeq2000 instrument following the manufacturer’s protocol and generate paired-end data (51 bp reads).

### Transcriptome assembly and functional annotation

The raw reads were first processed using the ShortRead package (R software) [16]. Sequence adapters and homopolymeric reads (with 45 or more of the same base) were removed. Ambiguous and low-quality reads (more than 10 bases with an Illumina quality score Q < 20) were also removed. Unpaired-end reads resulting from the previous trimming steps were discarded. Finally, only reads longer than 30 nucleotides were considered for the assembly process. Assembly was performed using Velvet version 1.0.07 [17] and Oases version 0.1.12 [18] software based on de Bruijn graphs using 41 nucleotides as optimal k-mer value. All data from this study have been deposited at the NCBI Sequence Read Archive (SRA) under BioProject PRJNA284566. *De novo* gene prediction was then performed on assembled transcripts using the Augustus web server [19] with *Tribolium castaneum* as training model. Corresponding amino acid sequences were annotated with metabolic information from the Kyoto Encyclopedia of Genes and Genomes (KEGG) using BlastKOALA [20, 21] to identify involved metabolic pathways. Gene identification parameters and accession numbers can be found in Additional file 1: Table S2.

### Differential gene expression analysis

To assess changes in gene expression in response to symbiosis, pairwise comparisons have been carried out between D1 and D6, and between D6 and D9. Only loci with a length > 250 nucleotides were considered for this analysis. This filter was shown to improve the correlation between replicates. Differential expression analysis was performed with the R package DESeq [22] by using a negative binomial distribution model. Dispersion values were estimated with the parameters: method = pooled, sharingMode = fit-only. An adjusted p-value for multiple testing was computed with the Benjamini-Hochberg procedure to control false discovery rate (FDR). Results with a FDR < 0.05 were considered statistically significant.

### Total RNA extraction and reverse-transcription

Total RNA from the gut tissue was extracted with the Trizol reagent (Invitrogen). RNA was incubated with 1 U/μg of RQ1 RNase-free DNase for 30 min, at 37 °C. The total RNA of guts was extracted using RNAqueous®-Micro (Ambion), which allows for a better RNA yield from small tissue samples. After purification, the RNA concentration was measured with a Nanodrop® spectrophotometer (Thermo Scientific) and RNA quality was checked using agarose gel electrophoresis. Reverse-transcription into the first strand cDNA was carried out using the First Strand Synthesis System for RT-PCR kit (Invitrogen).

### Real-time RT-qPCR transcript quantification

The quantification was performed with a LightCycler instrument using the LightCycler Fast Start DNA Master SYBR Green I kit (Roche Diagnostics). Primers were designed to amplify fragments of approximately 200 bp. A complete list of the primers can be found in Additional file 2: Table S1. The PCR reactions were carried out in LightCycler 96-well plates in a final volume of 10 μl, containing 2.5 μl of cDNA samples (diluted fivefold) and 7.5 μl of Light Cycler® 480 SYBR Green Master 1 mix, with 0.5 μl of 10 mM of each primer, 1.5 μl H2O and 5 μl of Mastermix. After 5 min, at 95 °C, the cycling conditions were as follows: 45 cycles at 95 °C for 10 s, 56 °C for 20 s and 72 °C for 30 s. Reactions were terminated by a quality control melting curve and a cooling. For each individual sample, the crossing point and the concentration of the gene transcripts were determined. Data were normalized using the ratio of the target cDNA concentration to that of two housekeeping genes: *glyceraldehyde 3-phosphate dehydrogenase* (*gapdh*) and *ribosomal protein L29* (*rpL29*). The plots represent the mean of 5 replicates for each point and error bars represent the standard error calculated as $\sigma /\sqrt{n}$ where σ is the sample standard deviation and *n* the sample size. For statistical analysis, a Generalized Linear Model with Gamma/link inverse transformation has been fitted to the data and analyzed using a two-way ANOVA with Chi-square test. P-values were considered significant if <0.05 for the factors “symbiotic status” and “time” and for the interaction. All statistical results can be found in Additional file 3: Table S3.

### Fluorescence in situ hybridization (FISH)

FISH experiments were performed on dissected symbiotic and aposymbiotic midguts. Eight to ten midguts for each experimental condition were fixed in PBS buffer with 4 % paraformaldehyde, embedded in paraffin, cut, and then mounted on poly-lysine coated microscope slides. After methylcyclohexan de-waxing and dehydration, sections were covered with a drop of 70 % acetic acid for permeabilization. Deproteinization of slides was performed in hydrochlorid acid 0.01 N with pepsin 0.1 mg/ml for 10 min at 37 °C. The sections were then pre-hybridized, hybridized with a *S. pierantonius*-specific 5’-end TAMRA-labeled oligo-probe targeting 16S RNA (TAMRA-ACC-CCC-CTC-TAC-GAG-AC-3’, 10 μg/mL), washed, and then mounted in PermaFluor™ Aqueous Mounting Medium (ThermoScientific) containing 3 μg/ml of 4’,6-diamidino-2-phenylindole (DAPI), as previously described [23].

Images were acquired with an epifluorescence microscope (Olympus IX81 equipped with a HQ535/50 filter for green signal, D470/40 for blue signal and HQ610/75 for red signal) and captured using an F-ViewII camera and the CellF software (Soft Imaging System). Images were treated and analyzed using ImageJ (release 1.49).

## Results and discussion

### RNA sequencing (RNAseq) libraries analysis

To unravel genes and cellular processes involved in symbiont dynamics, we have constructed six RNAseq libraries from adult weevil midguts at day 1 (D1), day 6 (D6) and day 9 (D9) after the UIM, with two biological replicates per modality. These time points were chosen in accordance with the endosymbiont recycling dynamics previously described by Vigneron et al. [13]. D1 represents a situation where endosymbiont population increases drastically. It then culminates at D6, before being eliminated during the next 8 days. Regarding D6, we assumed that the transcriptomic processes involved in the endosymbionts elimination are already setting up. Similarly, we presumed that the process of elimination is particularly intense between D6 and D9, when most of endosymbionts are eliminated and the insect has achieved its development.

After quality control and trimming of the raw reads, library size ranged from 27 to 48 million reads. Assembly resulted in a midgut reference transcriptome of 61 776 transcripts exceeding 250 bp, of which 56 % were identified and annotated. To determine whether the RNAseq replicates were reliable, we performed a correlation analysis based on a linear regression model between both replicates. The regression analysis resulted in an adjusted R-squared of 0.9527 (*p*-value <2.2 × 10^−16^), attesting a high degree of reproducibility. As this was the first time a high-throughput RNAseq experiment was performed on *S. oryzae*, we also compared the RNAseq expression values with experimental RT-qPCR transcript level values for some representative genes (Additional file 4: Figure S1; list of tested genes and primers in Additional file 2: Table S1). The regression analysis resulted in an adjusted R-squared of 0.8343 (F, 162.1; d.f., 31; *p*-value = 7.51 × 10^−14^) and showed a high degree of reliability for the RNAseq results.

### Analysis of differential gene expression and KEGG terms

**Fig. 1:**
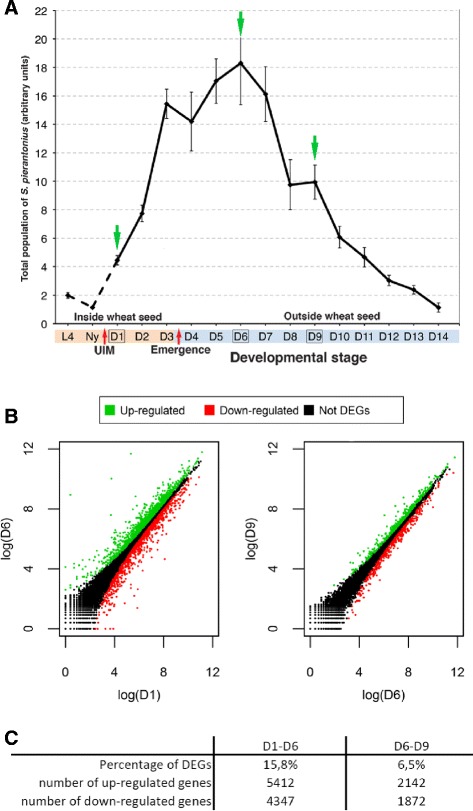
Analysis of Differentially Expressed Genes (DEGs) between Day 1, Day 6 and Day 9 after emergence. **a** qPCR quantification of endosymbiont DNA from 4th instar larvae (L4), late pupae (Ny), and adult males (Dx). Red arrows represent the UIM (Ultimate Insect Molting) and the emergence of the young adult from the wheat grain. Green arrows highlight the time points chosen for the RNAseq analysis. Adapted from Vigneron et al., [13]. **b** DEGs between D1 and D6 and between D6 and D9. The analysis shows a large proportion of transcriptional changes (up- and down-regulations) for the D1-D6 comparisonRed: significantly down-regulated genes; green: significantly up-regulated genes; black: non-differentially expressed genes. **c** Number and proportion of genes that are significantly differentially expressed between D1 and D6 and between D6 and D9

Pairwise comparisons were performed to identify Differentially Expressed Genes (DEGs) from D1 to D6, and from D6 to D9 (Fig. 1). With 5,412 up-regulated and 4,347 down-regulated genes, the D1-D6 comparison yielded the majority of DEGs compared with the D6-D9 comparison that produced 2,142 and 1,872 genes up- and down-regulated, respectively. Such a result highlights that most of the transcriptional changes related to symbiosis and development occur between D1 (symbiont burst) and D6 (utmost endosymbiotic load). The overall D6 transcriptional profile is also much more similar to the D9 than to the D1 profile, indicating that transcriptional regulations driving the endosymbiont elimination are already ongoing at D6, i.e. before symbiont elimination is detectable by q-PCR.

**Fig. 2:**
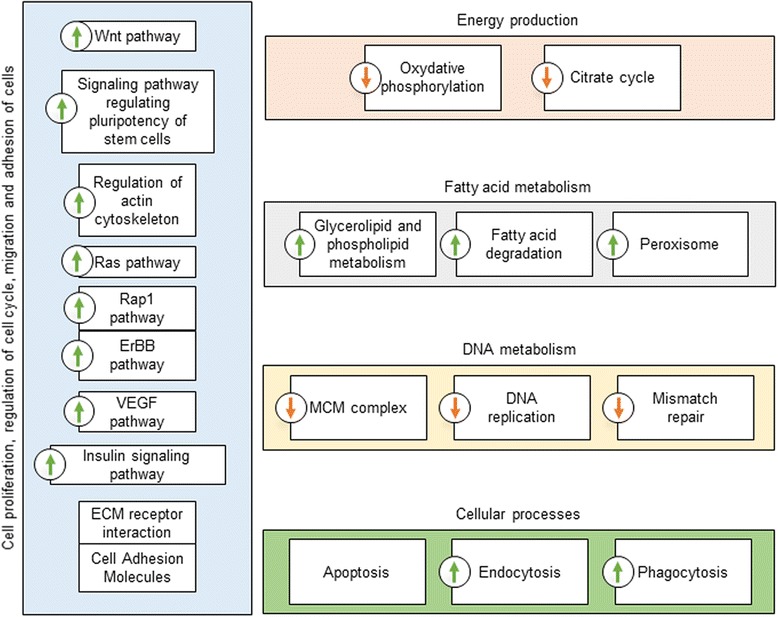
Main KEGG terms regrouping DEGs from D1 to D6. Are described each KEGG pathway from which at least five sequences are differentially expressed from D1 to D6. Terms were manually grouped into the five functional categories. The arrows indicate whether the pathway is globally overactive or underactive in D6 when compared to D1 (green up arrow: most of the term-attached sequences are up in D6; red down arrow: most of the term-attached sequences are down in D6; no arrow: a similar number of term-attached sequences are up or down in D6, allowing no reliable interpretation about the term activity changes)

In order to generate a transcriptome annotation allowing the detection of the metabolic and signaling pathways involved in endosymbiont elimination, we have conducted a KEGG-based analysis. Protein sequences corresponding to DEGs in both D1-D6 and D6-D9 comparisons were mapped to the KEGG database. Such an approach allowed identifying a set of 253 different KEGG terms displaying a different activity between the three time points. This list has been manually curated to identify and discard irrelevant terms because of i) their specificity to other taxa (e.g. mammalian “Adaptive Immunity” or “Bacterial Secretion Systems”); ii) the low number of associated DEGs; and, iii) the identification of unspecific DEGs such as general signaling key components that are involved in multiple pathways. The manual curation resulted in 21 KEGG terms with a different activity in D1-D6, and only 4 terms in D6-D9. In order to cluster closely related terms, the 21 D1-D6 differential terms have been manually classified into 5 functional categories: *Cell Proliferation and Regulation of Cell Cycle*, *Energy Production*, *Fatty Acid Metabolism*, *DNA Metabolism* and *Other Cellular Processes* (Fig. 2). The 4 D6-D9 differential terms are *Ras Pathway*, *Phospholipid Metabolism*, *Phagocytosis* and *Lysosome*.

The “energy production” KEGG class decreases from D1 to D6, which might be explained by two non-exclusive hypothesizes: the oxidative phosphorylation and citrate cycle are more active in D1 in relation to the high energy demand for developmental processes paralleling symbiont multiplication; the host energy production at D6 decreases as a result of the high energy income from symbiont digestion. As the host starts recycling symbionts through autophagy, energy becomes massively available from bacterial components digestion. The host energy production systems become under-solicited and decrease their activity. We hypothesize that the “DNA metabolism” KEGG class is linked to the midgut development and bacteriome differentiation at the apex of the mesenteric caeca. This process is reasonably more important at D1, when the midgut is under development, than at D6 when most of the tissues are in place. Along with metabolic changes, most of the differential KEGG terms seem related to development and to cellular mechanisms that are directly involved in the symbiont elimination. Among these processes are notably “apoptosis”, and autophagy, through the KEGG terms “fatty acid degradation”, “peroxisome” and “phagocytosis”, representing the autophagolysosomal degradation of bacterial components.

**Fig. 3:**
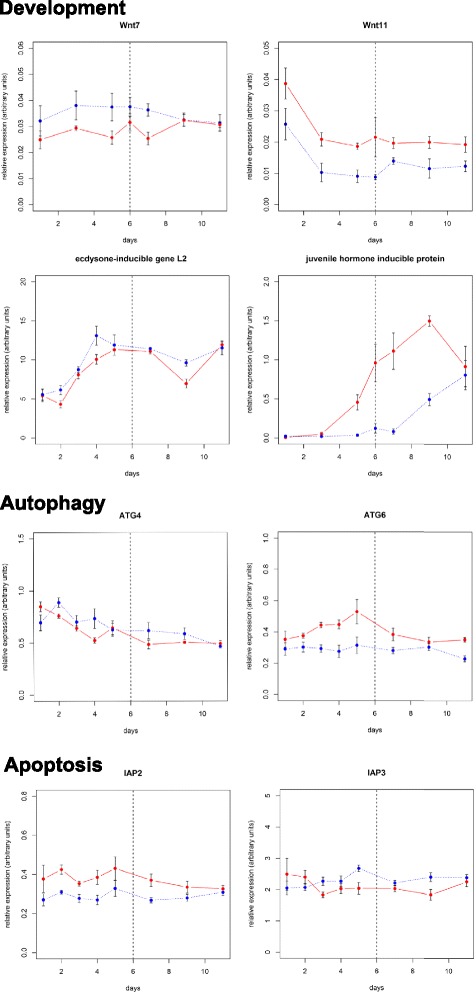
Kinetics of expression of developmental, apoptosis- and autophagy-related genes identified through the RNAseq analysis. Transcript level quantification has been performed both on symbiotic (red, plain line) and aposymbiotic (blue, dotted line) individuals. Each point represents the mean of five replicates, and the error bars represent the standard errors. The central dotted line on each plot symbolizes D6, when the endosymbiont dynamics switches from a bursting increase to a decrease due to their host-controlled recycling

Altough these RNAseq data give a global insight on pathways that may be differentially active at different times of the gut development, they do not discriminate between processes involved in symbiont dynamics and processes involved in midgut intrinsic development. To differentiate these two functions, we monitored the transcript levels of genes representative of each pathway in both symbiotic and aposymbiotic insects using RT-qPCR (Fig. 3). We chose to focus on the Wnt/wingless pathway, which is one of the most represented developmental pathway in the “Cell Proliferation” KEGG category. The Wnt/wingless pathway is required for a broad range of developmental processes [24]. The pathway is activated by the recognition of the Wnt ligands by the Frizzled receptors. Its activation leads to the release of β-cathenin/Armadillo that binds to the transcription factor Pangolin/TCF (T-Cell Factor), which regulates target gene expression. Nine types of Wnt proteins have been discovered in *Tribolium castaneum*, a coleopteran species closely related to *S. oryzae*; each Wnt impacts differently the insect’s development [25]. The analysis of *S. oryzae* RNAseq revealed two Wnt coding sequences, *wnt7* and *wnt11* that are differentially expressed along the symbiotic dynamics and between symbiotic and aposymbiotic weevil midgut (Fig. 3). *wnt7* transcript steady state level is lower in symbiotic than in aposymbiotic insects from D1 to D9 (*p*-value = 0.0005), and then reaches similar value from D9 to D11, when symbionts are in the way of a complete elimination. *wnt11* shows an opposite expression pattern, being more highly expressed in symbiotic than aposymbiotic insects (*p*-value = 1.10^−9^). *T. castaneum wnt7*, homologous of *D. melanogaster wnt2*, is involved in tracheal development [26], genital disc development [27] and attachment of flight muscles to the epidermis [28]. *wnt11* function is unknown in insects. In human, a *wnt11* homologue was shown to be required for progenitor and stem cell development [29, 30], and also influences tumoral cells proliferation [31, 32], pointing to a proliferation-regulator function. Although *wnt7* and *wnt11* gene functions have not been investigated in weevils, we hypothesize that they may interact with cell processes paralleling bacteriocyte differentiation and endosymbiont multiplication. These data suggest that the Wnt/wingless pathway may be differentially regulated in symbiotic individuals through the altered expression of Wnt genes.

**Fig. 4:**
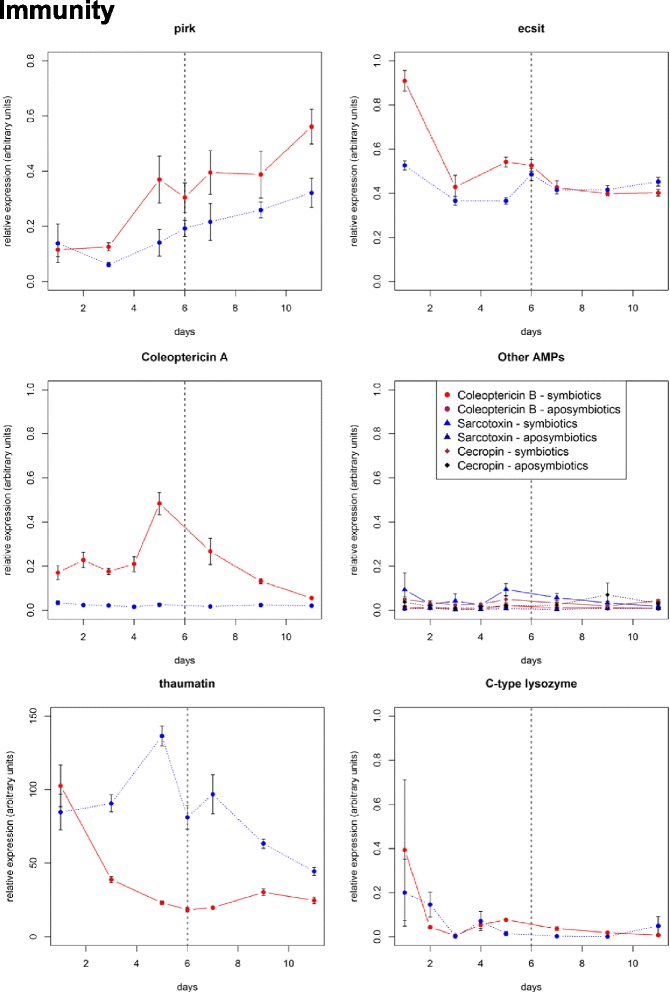
Expression of immune-related genes in symbiotic and aposymbiotic individuals. Transcript level quantifications have been performed both on symbiotic (red, plain line) and aposymbiotic (blue, dotted line) midguts. Each point represents the mean of five replicates, and the error bars represent the standard errors. The central dotted line on each plot symbolizes D6, when the endosymbiont dynamics switches from a bursting increase to a decrease due to their host-controlled recycling

Hormonal regulation through ecdysone and juvenile hormones is known to be involved in the development of young adults in many insect species, therefore we expected the high throughput analysis to yield DEGs and pathways related to hormonal activity. However, the number of DEGs associated with hormone-related processes was too low to generate a whole KEGG class. To assess the involvement of hormones in development and endosymbiont dynamics, we thus analyzed the expression of genes under the transcriptional control of ecdysone (*ecdysone-inducible gene L2*, *eigl2*) and juvenile hormone (*juvenile hormone inducible protein*, *jhip*) (Fig. 4). Interestingly, *eigl2* expression changes during insect development (*p*-value = 1.10^−6^), but its transcript level remains similar between symbiotic and aposymbiotic insects (*p*-value = 0.07742), suggesting that the symbiotic status does not interfere with ecdysone activity. On the other hand, *jhip* expression strongly increases in symbiotic insects from D3 to D9 (p-value < 10^−16^) while it remains low in aposymbiotic insects. In the latter, the expression of JHIP increases only from D9, showing a delay in their hormonal activity compared to symbiotics. The juvenile hormone has a variety of functions in emerging adults, including imaginal development and reproduction [33]. This work indicates that juvenile hormone also interacts with endosymbiont dynamics in two possible ways: the symbiotic burst could trigger an increase in juvenile hormone activity through such an unknown mechanism; the juvenile hormone activity may accelerate the symbiotic burst. In both case, the increase in juvenile hormone activity may participate in the accelerated development of symbiotic insects.

### Apoptotic and autophagic processes driving symbiont elimination are set-up during the symbiotic burst

It has been shown recently that one-week-old adult weevils recycle endosymbionts through apoptosis and autophagy [13]. Autophagy would allow the insect to digest the endosymbionts and to redirect their derivatives to its metabolism. The RNAseq analysis displayed this process in the D1-D6 differential KEGG pathways dataset. As pointed above, the increased fatty acid metabolism may reflect the digestion of symbiont membranes that were shown to accumulate as lamellar bodies during symbiont recycling [13]. To strengthen these results, we have monitored the expression of AuTophaGy-related genes 4 and 6 (*atg4* and *atg6*, Fig. 3), involved in the regulation of autophagy in *D. melanogaster* [34–36]. *atg4* showed a slightly increased expression in aposymbiotic insects compared to symbiotics (*p*-value = 0.01387), but the same expression kinetics in both types of insects (interaction *p*-value = 0.44290), suggesting no biological function in symbiosis-related autophagy control. *atg6* however showed a higher expression in symbiotic than aposymbiotic insects (*p*-value = 6.10^−13^) (Fig. 3). *atg6* expression does not vary upon time in aposymbiotic midguts. In symbiotics, its expression parallels symbiont dynamics, with an increase from D2 to D6 and a decrease from D6 to D11 (p-value symbiotic vs aposymbiotic insects = 6.10^−13^). Considering the latency between gene expression and its actual physiological effects, we assume that the expression increase from D2 to D6 is the early transcriptional set-up for the autophagic digestion of symbiont from D6 onward.

We also have monitored the expression of genes encoding Inhibitor of APoptosis 2 and 3 (*iap2* and *iap3*) [37, 38]. *iap2* and *iap3* genes have been described previously as strongly expressed in the weevil larval bacteriome under physiological conditions [39], suggesting their involvement in the bacteriome cellular homeostasis through the inhibition of apoptosis. In the adult midgut, *iap2* is more expressed in symbiotic that in aposymbiotic individuals from D1 to D9 (*p*-value = 1.10^−13^) (Fig. 3), suggesting its implication in symbiosis maintenance from D1 to D6, at least. The *iap3* gene does not show any differential expression, neither between symbiotic and aposymbiotic insects (*p*-value = 0.51013), nor in the course of time (*p*-value = 0.14220). At the transcriptional level, autophagy and apoptosis do not seem to be concomitant although they both participate in symbiont recycling. In the coral-dinoflagellates symbiosis, host cells first induces autophagic self-digestion under violent stress conditions, and switches to apoptosis when the stress reaches a certain threshold level [40]. In *S. oryzae*, the same sequential activation seems to happen, but as a genetic encoded process independent of environmental stress. We propose that autophagy is first triggered and is involved in the recovery of the major part of the symbiont structural components. Then, as the symbiont load becomes very low inside the cells, the triggering of apoptosis leads to the full elimination of the bacteriocytes and recovery of the last nutrient batch from the remaining symbionts, but also from their former housing cells that are no longer required.

### Endosymbionts recycling does not trigger antimicrobial peptide production

It has been shown that *S. pierantonius*, when artificially injected in the hemolymph of larvae, is recognized as an intruder and triggers the insect humoral immune activation and the production of antimicrobial peptides (AMP) [3]. The weevil midgut has also been shown to generate a local immune response upon exposure to exogenous bacteria, leading to the induction of AMP coding genes [41]. Thus, it has been speculated that the midgut immune response might be triggered following potential bacterial externalization from the bacteriome during endosymbiont dynamics. Surprisingly, very few immune-related genes were identified as differentially expressed along with endosymbiont dynamics in the RNAseq analysis. In the raw differential expression data, the two highest differentials for immune-related genes were *pirk* (*poor imd response upon knock-in* [42]*,* also referred to as *PIMS* (*PGRP-LC-interacting inhibitor of Imd signaling*) [43] and *rudra* [44]) and *ecsit* (*evolutionary conserved intermediate in the Toll pathway)* genes. Pirk is a negative regulator of the IMD pathway involved in the modulation of the immune response to Gram-negative bacteria and in the tolerance of gut-associated microbiome in *D. melanogaster* [42–44]. Remarkably, *pirk* transcript level is similar in symbiotic and aposymbiotic midguts at D1, but is significantly increased in symbiotic insects from D3 to D11 when compared to aposymbiotic insects (*p*-value = 2.10^−6^; Fig. 4). Ecsit is a signal transduction protein, involved in the Toll-like Receptor (TLR) pathway in vertebrates and Toll pathway in *D. melanogaster* [45, 46]. Its transcript level was higher in symbiotic than in aposymbiotic midguts from D1 to D5 (p-value <10^−16^), then decreased to reach a similar level (Fig. 4). This down-regulation of an immune signaling intermediate (i.e. *ecsit*) paired with the up-regulation of a negative immune regulator (i.e. *pirk*), points to a global decrease of the midgut immune signaling and presumably to a negative control on AMP production. To strengthen this finding, we have monitored by RT-qPCR the expression of AMP coding genes, which were not detected in the high throughput analysis. We have analyzed the expression of four AMP coding genes, namely *coleoptericin A, coleoptericin B, sarcotoxin and cecropin. coleoptericin B, sarcotoxin and cecropin* were identified from previous studies and are considered to be representative of the immune response of *S. oryzae* [41]*.* Coleoptericin A was shown to target weevil endosymbionts and to limit their infection to the bacteriome organ, by inhibiting bacterial cell divisions [47]. The transcription of the three other AMP-encoding genes is strongly up-regulated following insect infections with pathogens [3, 39, 41, 47]. In this study, *coleoptericin B, sarcotoxin and cecropin* showed no significant expression changes with time, remaining at the basal expression level in both symbiotic and aposymbiotic midguts (respective p-values for *coleoptericin B*, *sarcotoxin* and *cecropin* are 0.9293; 0.1616; 0.3622; Fig. 4). Remarkably, however, the expression profile of the *coleoptericin A* in symbiotic midguts parallels endosymbiont dynamics, with an increase from D1 to D6 and a decrease from D6 to D11 (*p*-value = 0.04327; Fig. 4). In addition, the *coleoptericin A* expression is not detected in midguts isolated from aposymbiotic individuals, suggesting the specificity of this AMP function with regards to symbiont control and maintenance.

In addition to these well-described AMP, we found one differentially expressed sequence in the D1-D6 dataset that matches a *thaumatin-like* gene. A *thaumatin* sequence was identified earlier in *S. oryzae* but has never been functionally studied [3]. Thaumatins were described as anti-fungal proteins with a glucanase function [48], although their precise mechanism of action is still unknown. In the soybean, a Thaumatin-like protein encoding gene is constitutively expressed in root nodules, allowing the plant to tolerate some strains of nitrogen-fixating symbionts [49]. The Thaumatin prevents bacteria from other strains to invade the host epidermal cells and to settle in the root nodule. In *S. oryzae*, *thaumatin* is highly expressed at D1 in both symbiotic and aposymbiotic midguts, but its expression drastically decreases in symbiotics during the symbiont population burst from D1 to D6, and remains low from D6 to D11 (Fig. 4). In aposymbiotic individuals, *thaumatin* expression is high and stable until D6, and decreases afterwards. We speculate that *thaumatin* down-regulation might prevent damaging the endosymbionts as their population increases. In combination with *pirk* and *ecsit* expression profiles, these results show that not only the gut immune response remains down-regulated during the whole endosymbiont dynamics changes, but also strongly suggest that immune regulators actively clamp AMP production. Taken together, these findings indicate that endosymbiont elimination and recycling is not preceded by AMP-mediated bacterial lysis, but rather relies on autophagy and apoptosis only. These cellular processes, by maintaining symbionts intracellularly, likely prevent inflammation and the release of microbe-associated molecular patterns in the hemolymph.

**Fig. 5:**
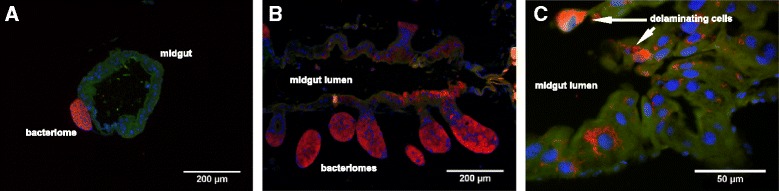
**a** Larval bacteriome showing *S. pierantonius* (red) secluded inside bacteriocytes. **b** Overview of an adult midgut showing *S. pierantonius* in bacteriocytes, epithelia and delaminating cells. **c** Delaminating epithelial cells in an adult weevil midgut, showing *S. pierantonius* in their cytosol

To check whether these mechanisms did efficiently maintain endosymbiont intracellular localization, we conducted Fluorescence In Situ Hybridization (FISH) experiments on larval and adult stages. In larval stages, endosymbionts remain strictly isolated within the bacteriome and no bacterium could be seen elsewhere, including in the gut epithelium (Fig. 5a). In D1, D6 and D9 adults, however, many endosymbionts were seen in other cells than bacteriocytes, and with a high density in several gut epithelial cells. Importantly, endosymbionts were never seen extracellularly (Fig. 5c). Delamination of epithelial cells into the gut lumen was also observed, including for symbiont-containing epithelial cells (Fig. 5c). Delamination and renewal of epithelial cells is a system of protection of the midgut integrity when facing bacterial infection or damages [50]. These observations suggest that delamination and likely epithelial renewal may be involved in the restructuration of the midgut along with the process of symbiont elimination by apoptosis and autophagy. Further analysis will be needed to address whether midgut epithelium delamination is associated with an increased cell renewal and whether it involves the Wnt/wingless pathway differential activity that has been highlighted in the KEGG analysis.

## Conclusions

This work provides new insights onto mechanisms involved in insect endosymbiont dynamics and control. The transcriptomic approach showed that weevil endosymbiont dynamics affects a broad range of cellular processes and pathways, including developmental pathways and metabolism of fatty acids and DNA. Genes involved in autophagy and apoptosis pathways are also activated before these cellular processes are detectable with PCR and histology. We also have demonstrated that the whole endosymbiont recycling process remains intracellular within the bacteriocytes and the midgut epithelium. Epithelial cells that contain endosymbionts undergo delamination, and are likely replaced through an increased epithelial cells renewal. Altogether these mechanisms prevent endosymbiont externalization from the bacteriocyte and epithelial cells and avoid immune activation and tissue inflammation. The host immunity pathways involved in AMP production remain clamped during the whole process, as attested by the low transcript levels of genes encoding AMP and the over-expression of negative immune regulators such as *pirk*. This regulation may allow the host to avoid a resource-expensive immune response that would clutter endosymbiont digestion, thus maximizing the nutrient recovery. *colA* gene transcript level exhibits a different profile than the other AMP, increasing in parallel with endosymbiont charge. Taken together, these findings provide evidence that insect endosymbiont regulation involves at least two main mechanisms: a first one that operates within the bacteriocytes and control symbiont cell division through “symbiosis-adapted” AMP (e.g. *colA* gene) [47], and a second one involving basic cellular processes, such as autophagy and apoptosis, that allow insects to control the symbiont charge in accordance to their physiological needs.

### Availability of supporting data

RNAseq data have been deposited at the NCBI Sequence Read Archive (SRA) under BioProject PRJNA284566. http://www.ncbi.nlm.nih.gov/bioproject/?term=PRJNA284566.

## Additional files

Additional file 1:
**Primers used in RT-qPCR experiments.** (XLSX 11 kb) Additional file 2:
**Characteristics of sequences used in RT-qPCR experiments.** (XLS 38 kb) Additional file 3:
**Detailed statistical analysis of RT-qPCR results.** (XLSX 26 kb) Additional file 4:
**Correlation analysis between RNAseq data and RT-qPCR data.** (TIFF 1054 kb)

## Abbreviations

AMP: AntiMicrobial Peptide
ATG: AuTophagy related Gene
Col: Coleoptericin
DEG: Differentially Expressed Gene
Dn: Day n
ECSIT: Evolutionarily Conserved Intermediate in Toll Pathway
EIGL2: Ecdysone-Inducible Gene L2
FISH: Fluorescent In Situ Hybridization
gapdh: glyceraldehyde-3-phosphate dehydrogenase
IAP: Inhibitor Of Apoptosis
IMD: IMmune Deficiency
JHIP: Juvenile Hormone Inducible Protein
KEGG: Kyoto Encyclopedia of Genes and Genomes
PIMS: PGRP-LC-interacting inhibitor of IMd Signaling
Pirk: Poor Imd Response upon Knock-in
NGS: Next-Generation Sequencing
qPCR: Quantitative PCR
RNAseq: RNA sequencing
rpL29: ribosomal protein L29
RT-qPCR: Real-Time Quantitative PCR
TCF: T-Cell Factor
TLR: Toll-like Receptors
UIM: Ultimate Insect Molting
