# Supplementary figures and images for: Weevil endosymbiont dynamics is associated with a clamping of immunity
Source: BMC Genomics. 2015 Oct 19;16:819. doi: 10.1186/s12864-015-2048-5 (PMC4617454; doi:10.1186/s12864-015-2048-5)

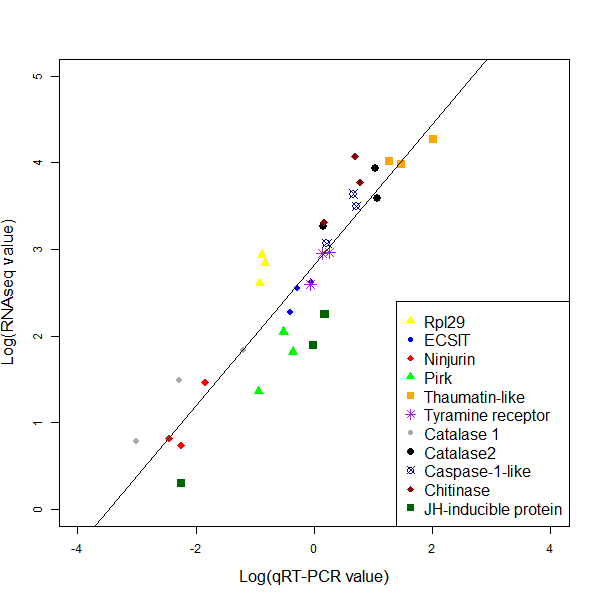

Supplement: Additional file 4: — Correlation analysis between RNAseq data and RT-qPCR data. (TIFF 1054 kb) [file 12864_2015_2048_MOESM4_ESM.tiff]
